# Extranodal Natural Killer/T-Cell Lymphoma: An Incidental Finding

**DOI:** 10.7759/cureus.1260

**Published:** 2017-05-19

**Authors:** Ashley Althoff, Michael Bibliowicz

**Affiliations:** 1 Medical Student, LECOM Bradenton; 2 Otolaryngology, Florida Hospital-Orlando

**Keywords:** extra-nodal nk/t-cell lymphoma, epstein-barr virus (ebv), smile regimen, nasal tumor, endoscopic sinus surgery, sinusitis

## Abstract

Extranodal natural killer/T-cell lymphoma (ENKTCL) is a rare form of non-Hodgkin lymphoma. This neoplasm is more prevalent in regions of Asia and Latin America and most commonly involves the sinonasal tract, presenting with signs of nasal obstruction, epistaxis, or sinus infection. It is a locally destructive and angioinvasive neoplasm. The treatment of ENKTCL is dependent on the extent of the tumor. For localized disease, the treatment is chemoradiation. For disseminated disease, treatment is mainly chemotherapy-based. This report describes a case of a 41-year-old Hispanic woman who initially presented with signs of nasal congestion for four weeks and was subsequently diagnosed and treated for chronic sinusitis. The patient underwent endoscopic surgery for persistent chronic sinusitis, with a presumptive diagnosis of allergic fungal rhinosinusitis based on clinical and radiographic presentation. The pathologic exam revealed a diagnosis of ENKTCL. The patient underwent three cycles of chemotherapy comprised of steroid (hydrocortisone), methotrexate, ifosfamide, pre-asparaginase, and etoposide (SMILE) followed by radiation, resulting in clinical and radiographic remission. On review of the literature, ENKTCL is very rare in the United States and diagnosis is commonly delayed due to non-specific signs. We report this case to increase awareness of this disease entity and remind clinicians to include this in the differential diagnosis of nasal obstruction.

## Introduction

Extranodal natural killer/T-cell lymphoma (ENKTCL), previously known as lethal midline granuloma, is a rare form of non-Hodgkin lymphoma. It comprises 7-10% of all non-Hodgkin lymphomas in Asians and Latin Americans but only 1% of Caucasians worldwide. ENKTCL most often involves the nasal cavity but rarely may also involve the gastrointestinal tract, skin, soft tissue, or testes. This neoplasm can take on the phenotype of either natural killer (NK) or T-cells and is virtually always Epstein-Barr virus (EBV)-positive. This tumor is locally destructive and invasive and carries a poor prognosis due to non-specific presenting signs that often delay diagnosis [1-3]. Current treatment regimens combine chemotherapy with radiation for localized disease or chemotherapy alone for more advanced disease [4]. In this study, we report a case of ENKTCL, nasal type, in a 41-year-old Hispanic woman that was incidentally discovered on biopsy for a presumed initial diagnosis of sinusitis. 

## Case presentation

A 41-year-old Hispanic woman presented to our otolaryngology office complaining of severe congestion associated with postnasal drainage, mucorrhea, and frontal headaches with photophobia and phonophobia for two month's duration. Additionally, she complained of loss of smell and taste. One month previously, she was treated empirically for chronic rhinosinusitis with a six-day pack of Sterapred, 5 mg, Keflex, 500 mg twice daily for two weeks, and Dymista (azelastine hydrochloride and fluticasone propionate), 137/50 mcg once daily.

On examination, the septum deviated to the right and turbinates were edematous. No palpable cervical lymphadenopathy was noted and the remainder of the head and neck exam was unremarkable. Nasal endoscopy revealed a polypoid mass in the region of the left nasal cavity and thick mucorrhea was noted.

**Figure 1:**
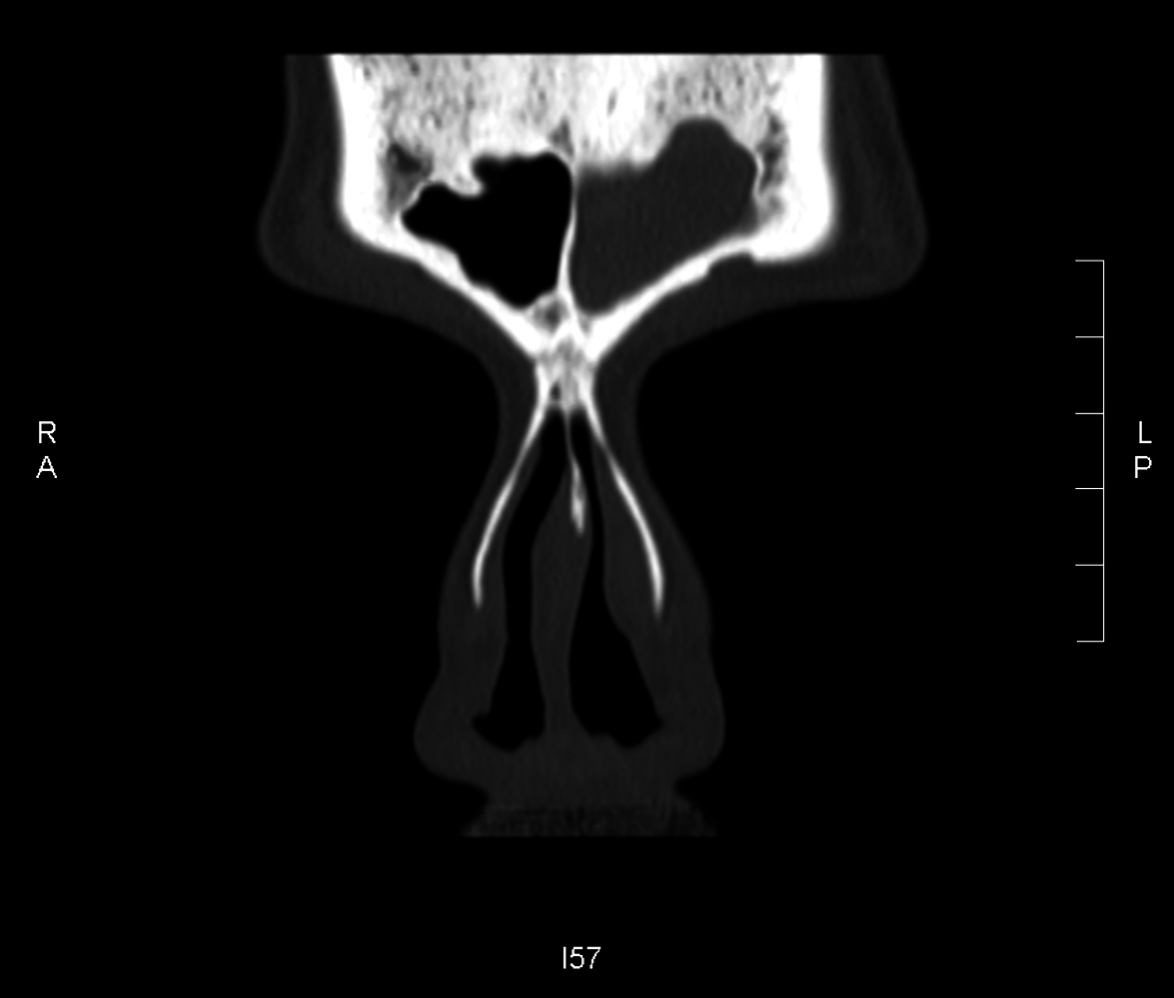
Complete opacification of the left frontal sinus

**Figure 2:**
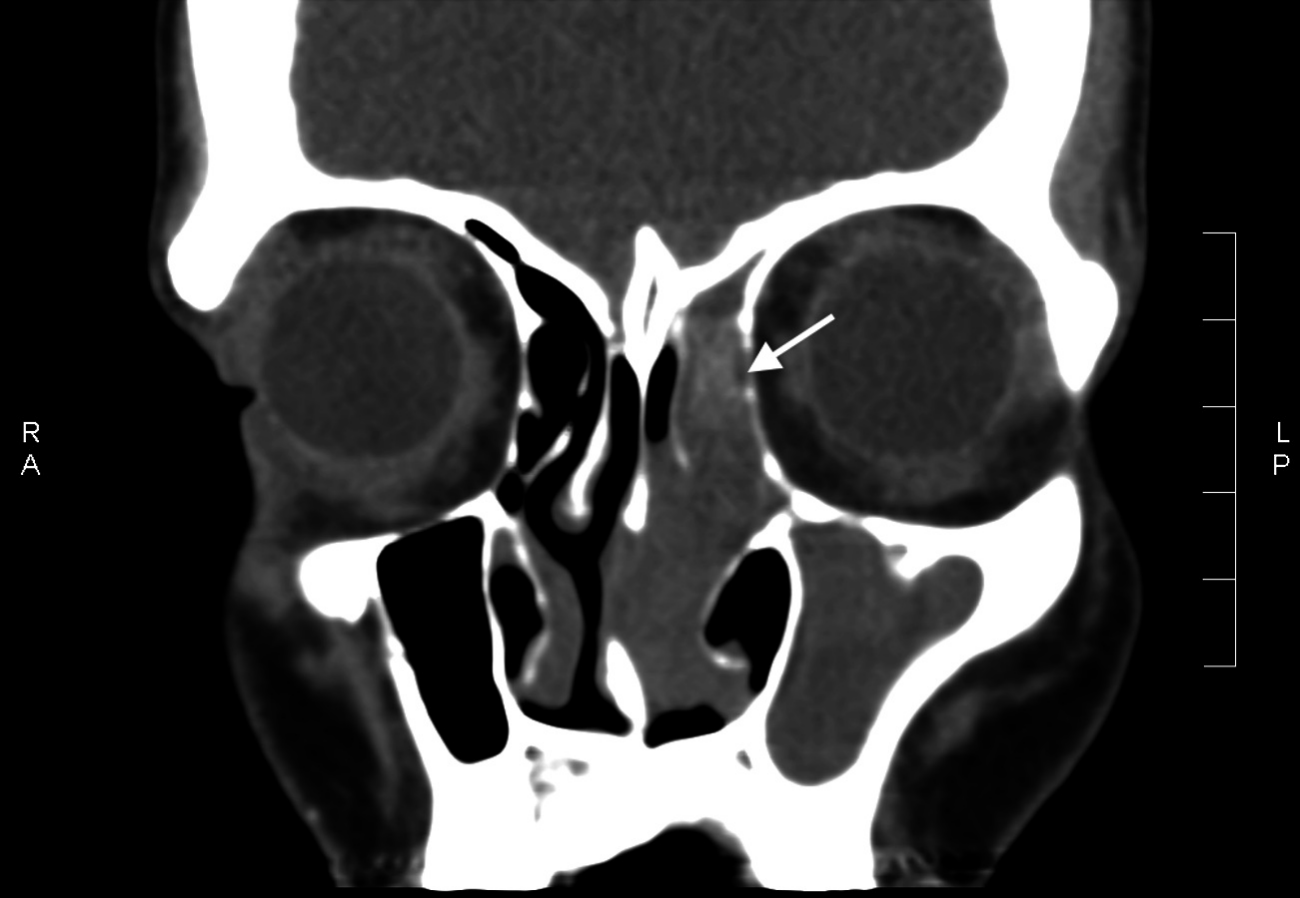
Opacification of the left maxillary sinus and nasal cavity with double density sign (arrow)

**Figure 3:**
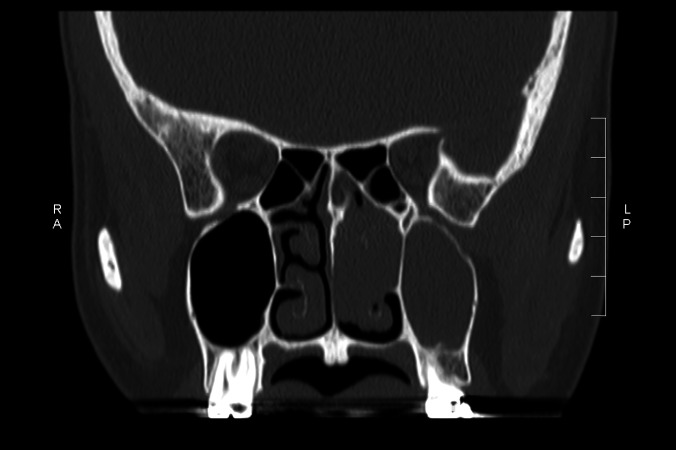
Extension of disease through nasal cavity into the nasopharynx

A computed tomography (CT) scan showed opacification of the left frontal sinus ( Figure 1), left maxillary sinus and left-sided ethmoidal air cells (Figure 2), and disease involving the nasal cavity and nasopharynx (Figure 3). A double density sign in the maxillary region was also seen and thought to be suggestive of allergic fungal rhinosinusitis (arrow in Figure 2).

Based on the CT scan findings and failure to respond to conventional treatment, allergic fungal rhinosinusitis was suspected. The patient was scheduled for left-sided image-guided endoscopic sinus surgery and started on 60 mg of prednisone for five days. Appropriate consent was obtained, and preoperative labs were unremarkable. 

The patient successfully underwent left-sided endoscopic sinus surgery consisting of a left frontal sinusotomy, left ethmoidectomy, and left sphenoidotomy with some resolution of the headache and congestion. Although allergic fungal rhinosinusitis was suspected initially, the intraoperative appearance of the tissue removed did not support this diagnosis. All specimens were sent to pathology for gram stain and cultures, which were negative.

**Figure 4:**
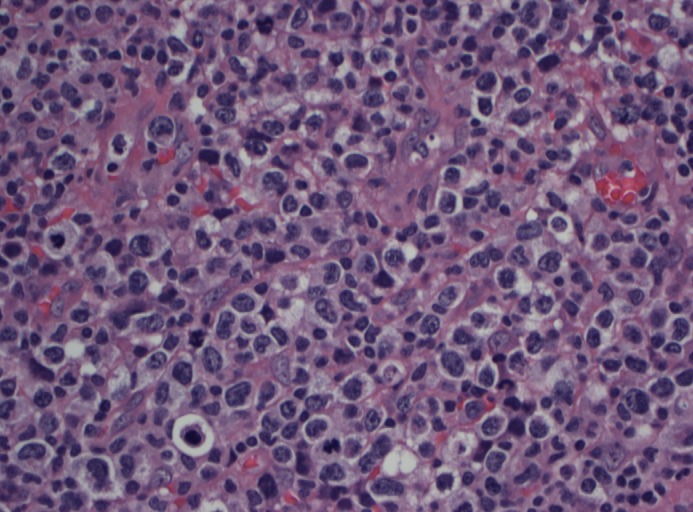
Large atypical cells with irregular nuclear contours, vesicular chromatin, multiple nucleoli, and clear cytoplasm completely effacing the lymph node architecture

**Figure 5:**
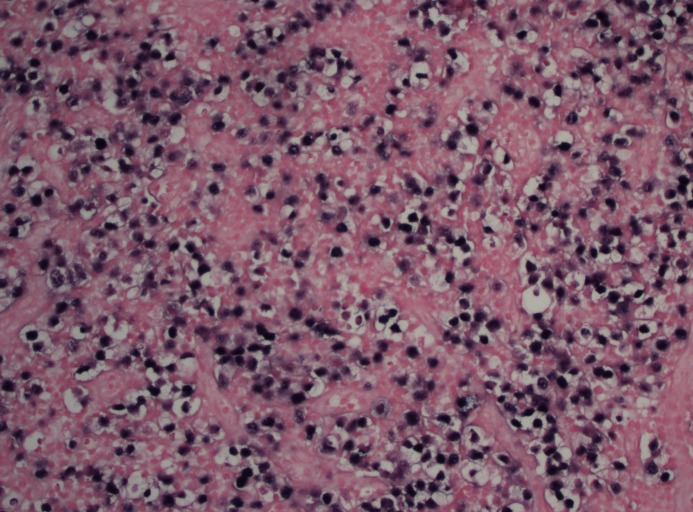
EBV by Epstein-Barr virus-encoded small RNAs in situ hybridization; cells are diffusely positive EBV: Epstein-Barr virus; RNA: ribonucleic acid

The histopathology report showed an atypical lymphocytic infiltrate of mostly large cells with an angiocentric pattern (Figure 4). Immunohistochemical stains were positive for CD45, CD3, CD7, CD2, BCL2, granzyme B, TIA-1, and EBV (Figure 5).  The stains were negative for CD56, CD20, CD79a, PX5, AE1/AE3, S-100, CD10, CD23, cyclin D1, CD34, TdT, CD30, CD4, CD8,  CD5, ALK-1, and programmed cell death protein 1 (PD1). These features were consistent with ENKTCL, nasal type.

**Figure 6:**
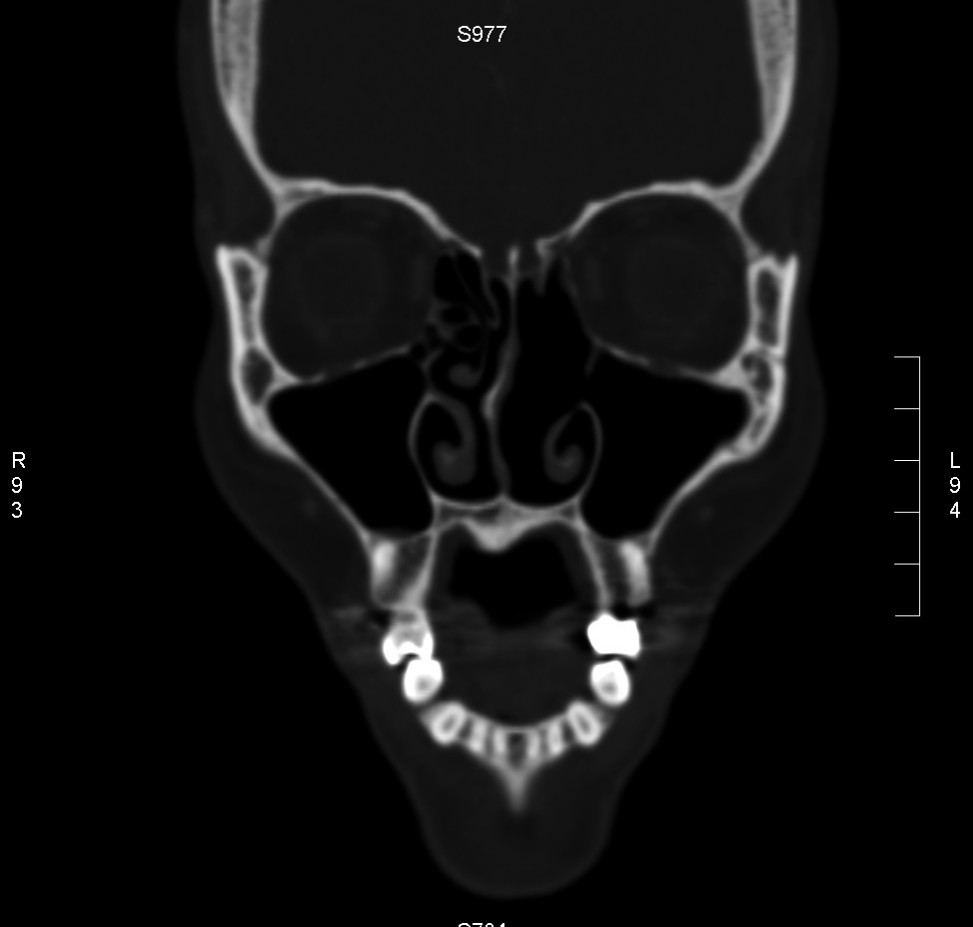
No residual disease noted after completion of treatment

Subsequently, the patient underwent further imaging for any evidence of distance metastases; positron emission tomography-computed tomography (PET-CT) and magnetic resonance imaging (MRI) were all unremarkable. One month after her diagnosis she was started on steroids (hydrocortisone), methotrexate with leucovorin, ifosfamide, pre-asparaginase, and etoposide (SMILE). She received three cycles of treatment followed by local radiation. The patient tolerated chemotherapy with the expected side effects of pancytopenia, drug-induced hepatitis, and hypotension. Biopsy of ethmoidal and maxillary sinus tissue after completion of two rounds of chemotherapy showed no evidence of lymphoma. PET scans at one-year post-treatment showed no evidence of lymphoma. Subsequent CT scans have also been negative for local recurrence (Figure 6).

## Discussion

ENKTCL is a rare form of non-Hodgkin lymphoma more common in the regions of China, Japan, Korea, Mexico, and Peru than in North America. ENKTCL most commonly involves the sinonasal cavity and has a 3:1 male to female ratio with a mean age of onset of 52. The etiology of this tumor is uncertain; however, it is known to have a strong association with EBV infection. The tumor is composed of mostly active NK cells and some cytotoxic T-cells [1, 3].

ENKTCL, nasal type, occurs in the upper aerodigestive tract involving the nasal cavity, nasopharynx, paranasal sinuses, hypopharynx, and larynx. The typical initial presentation is with non-specific symptoms, such as nasal obstruction, epistaxis, and facial swelling [2]. As in the case reported here, ENKTCL can mimic sinus infections, delaying treatment [1]. Therefore, a high index of suspicion must be adopted.

Flexible and rigid nasal endoscopy can be used to visualize the nasal cavity, and biopsies should be performed if there is any suspicion of a destructive pathology [1]. In order to distinguish ENKTCL from other destructive etiologies, neoplasms, and benign processes, large biopsies must be obtained from suspicious areas [5].

Histologically, ENKTCL shows an angiocentric pattern of growth with vascular destruction and necrosis. Tumor cells range from small to large and are dispersed among plasma cells, lymphocytes, eosinophils, and histiocytes. Flow cytometry and immunohistochemistry stains demonstrate the presence of T and NK cell markers (CD2, CD56, CD3), along with cytotoxic molecules, such as granzyme B, TIA-1, and perforin. Unlike T-cells, NK cells do not display surface CD3. The case reported was positive for CD3 and CD2, confirming T-cell origin, but negative for CD56. This neoplasm was unique in that it was more represented by T-cell phenotype as opposed to NK cells, the typical phenotype [3]. Expression of EBV ribonucleic acid (RNA) by in situ hybridization is necessary for diagnosis, which the patient indeed had [6].

On imaging, ENKTCL nasal type can be seen as diffuse mucosal thickening along the nasal turbinates or as a destructive midline mass. This can be difficult to distinguish from a fungal infection, Wegener’s granulomatosis, or other neoplasms [7]. CT is recommended to visualize the extent of the disease, MRI for visualization of soft tissue and bone involvement, and lastly, the use of PET-CT is recommended due to its accuracy and ability to differentiate between inflammation and tumor [2].

In the case reported above, the patient was suspected of having fungal sinusitis from the CT imaging. Tumor staging was not done prior to surgery; however, a PET-CT and MRI were performed after tissue diagnosis and were negative for local and distant disease.

The treatment for ENKTCL has evolved over the years. Previously, a cyclophosphamide, doxorubicin, vincristine, and prednisolone (CHOP) therapy was used with poor results due to NK cells displaying high levels of P-glycoprotein that confers resistance to anthracyclines [2]. The current recommended treatment is the SMILE protocol, which is more specific against NK cell neoplasms and is unaffected by the multidrug-resistant phenotype. For localized disease, local radiotherapy of at least 45 Gy is also recommended [4]. Following current recommendations, the patient was placed on three cycles of SMILE therapy followed by localized radiation.

ENKTCL is an aggressive neoplasm with a five-year survival of 40-59% following treatment. However, 30-40% of patients show relapse of the disease; most recurrences occur within the first two years [2]. The patient showed excellent response to treatment and remains disease-free at the three-year mark. 

## Conclusions

In summary, we describe a case of an aggressive, frequently lethal lymphoma, rarely seen in the United States in order to increase awareness and aid in the diagnosis. ENKTCL presents with non-specific signs and symptoms that can lead to misdiagnosis and delayed treatment, so practitioners must include this in their differential for suspected chronic sinusitis that does not respond to medical therapy. Current treatments include asparaginase-based chemotherapy regimens (SMILE) and radiation. If diagnosed early, this devastating disease can be treated successfully. 
